# Expert gaze as a usability indicator of medical AI decision support systems: a preliminary study

**DOI:** 10.1038/s41746-024-01192-8

**Published:** 2024-07-27

**Authors:** Nora Castner, Lubaina Arsiwala-Scheppach, Sarah Mertens, Joachim Krois, Enkeleda Thaqi, Enkelejda Kasneci, Siegfried Wahl, Falk Schwendicke

**Affiliations:** 1 Carl Zeiss Vision International GmbH, Tübingen, Germany; 2 University of Tübingen, Tübingen, Germany; 3 Charité – Univesitätsmedizin, Oral Diagnostics, Digital Health and Services Research, Berlin, Germany; 4 Technical University of Munich, Human–Centered Technologies for Learning, Munich, Germany; 5 Institute for Ophthalmic Research, University of Tübingen, Tübingen, Germany; 6 Ludwig Maximilian University, Operative, Preventative and Pediatric Dentistry and Periodontology, Munich, Germany

**Keywords:** Human behaviour, Interdisciplinary studies, Computer science

## Abstract

Given the current state of medical artificial intelligence (AI) and perceptions towards it, collaborative systems are becoming the preferred choice for clinical workflows. This work aims to address expert interaction with medical AI support systems to gain insight towards how these systems can be better designed with the user in mind. As eye tracking metrics have been shown to be robust indicators of usability, we employ them for evaluating the usability and user interaction with medical AI support systems. We use expert gaze to assess experts’ interaction with an AI software for caries detection in bitewing x-ray images. We compared standard viewing of bitewing images without AI support versus viewing where AI support could be freely toggled on and off. We found that experts turned the AI on for roughly 25% of the total inspection task, and generally turned it on halfway through the course of the inspection. Gaze behavior showed that when supported by AI, more attention was dedicated to user interface elements related to the AI support, with more frequent transitions from the image itself to these elements. When considering that expert visual strategy is already optimized for fast and effective image inspection, such interruptions in attention can lead to increased time needed for the overall assessment. Gaze analysis provided valuable insights into an AI’s usability for medical image inspection. Further analyses of these tools and how to delineate metrical measures of usability should be developed.

## Introduction

Even in an era where artificial intelligence (AI) is becoming more pervasive in the workforce, human experts are still sought-after for the final decision. In a wide range of fields, such “collaborative” AI-human systems are the preferred constellation: In education^1,2^, marketing^3,4^, semi-autonomous vehicles^5^, and ever-increasing in healthcare^6–8^. AI serves as support or assistant systems, while any final decision is taken by a human, fulfilling the demand for human oversight and autonomy^9^.

AI decision support systems for medical image interpretation – e.g., inspecting x-rays or volumetric scans – have been shown to improve diagnostic accuracy^10–12^. Collaborative AI that supports radiologist inspection has led to faster and more accurate diagnoses compared with experts or AI alone^11,13–16^. However, other studies show mixed results regarding performance and allude to concerns that AI can hinder expert diagnoses or offer little support^17–21^. These concerns can transfer to patients, who feel more assured when they know the expert has made the final diagnosis after seeking AI support^22,23^. Medical experts have also expressed apprehension using AI, regarding concerns of liability, trust and understanding, and reliability^24–27^. These concerns solidify AI as an assistant tool and not autonomously making decisions.

Though a wealth of peer-reviewed publications highlight the potential for medical AI support systems, very few systems are successfully adopted into daily clinical workflows (See overviews in refs. ^6,28–30^). There are a number of factors that are holding back its integration into clinical environments, like regulatory hurdles^31^, unclear diagnostics, efficacy, and usefulness^6,26^. Others suggest that critical limitations affecting integration of these systems are unsatisfactory experience and interoperability difficulties^32–34^. Given interaction problems between the human user and the AI system, trust in AI systems can be greatly impacted^35–37^.

For collaborative AI systems, ease of interaction is essential. So far, little effort has been directed towards improving the interaction between human expert and system. When AI interaction is uncomfortable for the user, trust in the system is diminished regarding the system’s performance and practicality^38^. Currently, the majority of research on AI medical support has focused on performance metrics, while ignoring practicality and interaction between the user and the system. Better interaction may improve usability and, subsequently, integration in professional workflows. If AI interactions are not deemed *useful*, these devices could be pushed aside in favor of human only workflows^34^. One approach to assessing interaction is expert gaze behavior.

Efficient and thorough inspection of medical images leads to faster feature recognition and better clinical reasoning^39–41^. The visual strategies of medical professionals are an interplay of heightened sensitivity to certain features or structures and prior knowledge, i.e., experience and case based. This context dependent gaze behavior is known as the scanpath, consisting of fixations (attentional information) and saccades (transitions between attentional areas)^42,43^.

Much of the previous literature in medical expert gaze analysis has focused on comparing experts and novices, highlighting the faster and more accurate perceptual ability of experts^44,45^: With shorter time to first fixation on relevant areas (e.g., an anomaly) than novices^40,46–52^. Experts also have more fixations and fixations of longer duration on relevant instead of irrelevant areas, which can be attributed to reducing extraneous attentional processing^44,53–56^. However, image content affects expert eye movements^40,44,57,58^. Obvious and easy to spot anomalies do not require as many fixations than harder to detect anomalies^40,58–63^. For further descriptions of gaze in the context of medical image inspection, see refs. ^39,64,65^.

Expert gaze and scanpath behavior have been investigated in the context of dental images, and depend on image type^66^. For panoramic radiographs, image coverage is key; outer and inner structural areas are assessed quickly, then thoroughly in a global to local search strategy^67,68^. For periapical radiographs, tooth-by-tooth and circular search strategies are preferred, depending on the nature of anomalies present^63^. Recently, this systematic tooth-by-tooth scanning strategy was also found when experts inspected bitewings for caries, which promoted faster recognition^69^. When anomalies are harder to detect, experts’ pupillary response indicates that their cognitive load adjusts to accommodate the information level^70^. This adaptability highlights experts’ effective information processing abilities. Whether this behavior can also accommodate information presented by a decision support system has yet to be explored.

Eye tracking has been used to evaluate the usability of systems in research fields such as marketing, software testing, and product design^71,72^. From an interaction perspective, eye tracking can address not only the *how* (e.g., how do they navigate the interface), but also the *why* (e.g., why is the image inspected in this way)^73,74^. Metrics such as fixation behavior and scanpath transitions and length related to interface elements can represent a user’s attention or understanding of taskflows^75,76^. These metrics have also been shown to correlate with usability reports^77,78^. Pupil diameter changes as an indicator of cognitive load can also indirectly assess usability^79^. Eye movement patterns can also indicate specific usability concerns, such as inconsistencies in design, architecture, and formatting^74^. This information can improve accessibility^80^, content highlighting^81^, and even realtime attention guiding^82^.

Eye tracking metrics have also been used as an indicator for usability of medical technologies^83,84^, for example, to assess the usability of intensive care ventilators^85^ or prosthetic arms^86^. Other studies have leveraged eye tracking findings to better design interfaces based on patient or clinician needs^86–88^. See Asan et al.^89^ for more literature on medical interface design from gaze research. Concerning AI decision support systems, eye tracking is a powerful tool in addressing system improvements. An array of research has used expert gaze to assist AI models with region segmentation and labeling^90–92^. These improvements can transform information visualization, which has shown to improve diagnostic performance^93,94^. In summary, eye tracking measures offer insight into system usability and can be collected during the task. However, these metrics have not yet been used to evaluate how experts integrate AI support into their own clinical decision-making strategies or how AI support could potentially interrupt their workflows.

The aim of the present study was to evaluate how experts interact with an AI-based decision support tool to investigate dental bitewings, i.e. radiographs used for detecting caries. We use gaze behavior analysis via eye tracking as a non-invasive, naturalistic, and objective measure of interaction. Eye tracking measures have been shown to be robust indicators of usability, which research on medical AI systems has yet to fully utilize. Also, expert gaze behavior during medical image examination is well understood, offering a link between gaze features and cognitive processes. We employed gaze behavior as an indicator of the visual strategies related to clinical decision-making when using AI support versus not using AI support. We hypothesized that gaze behavior when using AI support will be different from gaze behavior without AI support. We also investigated how experts interact with the AI support system in the context of how they incorporate components of the system as well as control the system, and how their interaction changed over the experiment.

## Results

### Statistical analysis

As we are concerned about factors related to usability, we offer no analysis related to how experts look at bitewings in the realm of clinical decision-making. Instead, we analyzed visual strategies in the context of how experts employ an AI-support software that presents a bitewing plus informational content as part of an interface. We group the informational content under the category of user interface (UI) elements. These parts of the interface are depicted in Fig. 3a. As scanpath behavior specifically linked to medical image content was out of the scope of this current research, we restricted our analysis to transitional eye movements around the software, which was presented in a web browser.

We report only fixational metrics, as the eye tracker sampling rate is too low to fully understand saccadic behavior. In addition to the gaze behavior between dentists with and without AI support, we provide descriptions of how they interact with the AI system: i.e., their mouse interactions and gaze behavior differences when the AI overlay is turned off and on.

All variables exhibited non-normal distributions and thus were summarized using median and interquartile range (IQR) and were analyzed using non-parametric tests. Differences in each gaze metric between relevant groups were tested using the Wilcoxon rank sum test, where level of significance was set to *p* < 0.05. Missing data was not imputed. To account for any possible spatial offsets in the gaze data, defined areas of interest (AOIs) were given an extra pixel padding based on their relative pixel area: A pixel padding of 3 degrees of visual angle. For fixation behavior analysis, we counted fixations that land in overlapping AOI as a hit in both AOI. All statistical analyses and data management were performed using Python (version 3.8 and above). Table 1 reports the results of the statistics.

**Table 1:** Summary statistics of fixation metrics based relative to the inspection task, attention to the bitewing and attention to user interface (UI) elements

	With AI support		Without AI support		
Variable	Med	IQR	Med	IQR	*p*-val
Task time [s]	109.19	[78.91, 140.78]	87.1	[73.37, 99.86]	0.0742
Total fixation duration UI [ms]	5481.76	[3820.35, 6966.61]	2348.39	[1071.496, 4963.75]	0.0003*
Total fixation duration bitewing [ms]	54393.53	[43855.15, 84542.02]	49139.55	[41812.34, 70003.19]	0.3168
Task average fixation duration [ms]	313.27	[243.597, 367.13]	317.29	[239.797, 349.86]	0.7660
Task fixation count	208.22	[182.6, 260.6]	182.70	[164.05, 212.39]	0.0548
UI fixation count	20.86	[18.83, 26.1]	10.63	[4.93, 15.65]	<0.001*
Bitewing fixation count	191.90	[161.8, 230.75]	176.55	[153.58, 204.25]	0.19
Task fixation frequency [fix/s]	2.21	[2.03, 2.33]	2.16	[1.98, 2.43]	0.8287

* indicates statistical significance at *p* < 0.05.

### Time on task

**Fig. 1:**
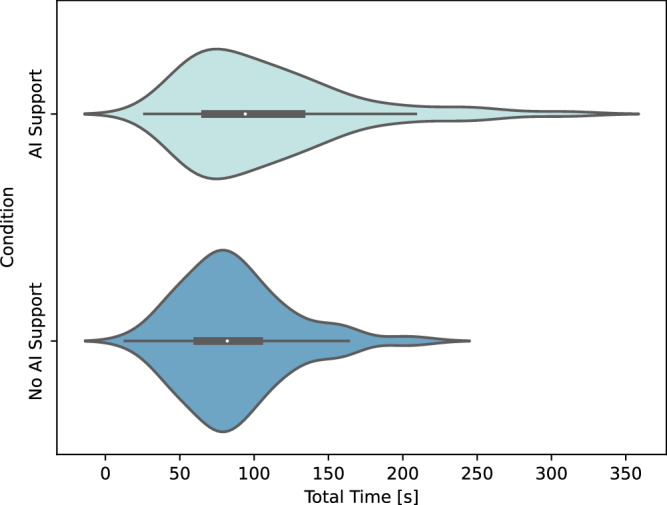
Total time on task, in seconds, for viewing bitewings with AI support and without. There are differing distributions between the two conditions, though their median task time is not significantly different.

**Fig. 2:**
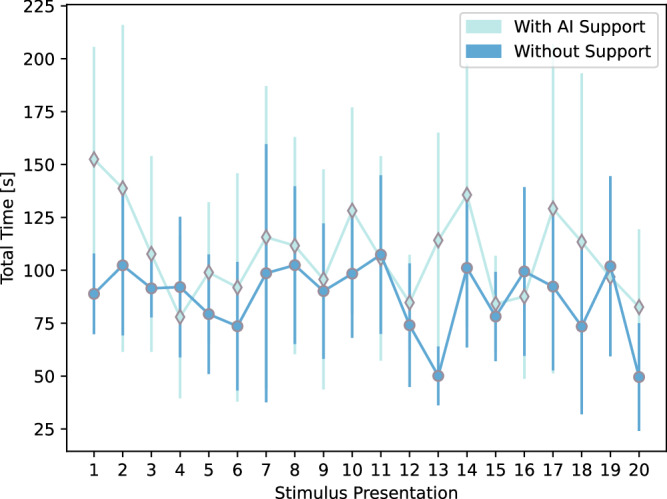
Task time for each image with and without AI support. We observe a slight effect of stimulus viewing order with the plotted task time for each image (means, with bars representing the standard deviation) with AI support (diamonds) and without AI support (circles).

We found that dentists took longer on the task when using AI support (109.19 s [78.91, 140.78]) than when not using AI support (87.1 s [73.37, 99.86]), while this difference was not significant given the wide spread of time taken (*p* = 0.0742). Figure 1 shows the distributions of task times for both conditions. We also observed a slight effect of stimulus viewing order with the plotted task time for each image (Fig. 2). For the first few images, there was a large difference in the inspection time between AI support and No AI support. This trend remains over the course of the experiment, but with slight inconsistencies.

Regarding the distribution of attention during the task, we normalized each participant’s time viewing the bitewing and viewing UI elements over their total task time to get a percent proportion. The proportion of time spent looking at the UI vs. looking at the bitewing showed that with AI support, total time viewing UI elements was doubled from 4.94 % to 8.4 %.

### Fixation behavior

Significantly more time was spent looking at the user interface when AI was present (5481.76 ms [3820.35, 6966.61]) than without (2348.39 ms [1071.496, 4963.75]: *p* = 0.0003). More time was spent viewing the bitewing with AI (54,393.53 ms [43,855.15, 84,542.02]) than without (49,139.55 ms [41,812.34, 70,003.19]), again without statistical significance, though (*p* = 0.3168). However, this behavior resulted in a higher number of total fixation counts with AI support (208.22 [182.6, 260.6]) compared to without AI support (182.70 [164.05, 212.39]: *p* = 0.05) Although, fixation count in the bitewing was not significantly different between AI (191.90 [161.8, 230.75]) and No AI (176.55 [153.58, 204.25] : *p* = 0.19), fixation count in the user interface (AI: 20.86 [18.83, 26.1], No AI: 10.63 [4.93, 15.65]) was significantly higher for the AI support condition (*p* < 0.001).

**Fig. 3:**
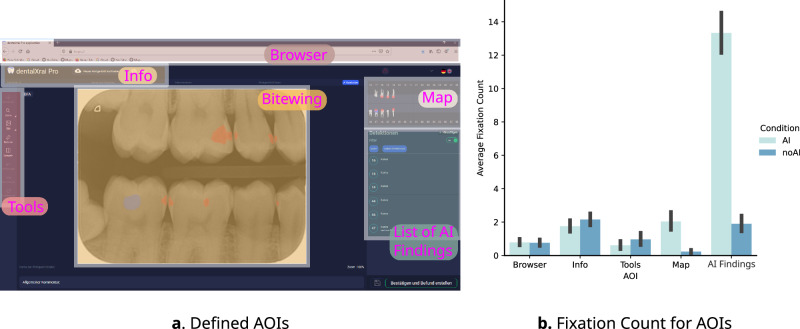
Visual attention to relevant regions (AOIs) of the AI support interface. **a** is a depiction of the stimuli used in the experiment, with the bitewing being in the center surrounded by user interface elements with the right-side elements related to the AI support, which were not visible in the no AI condition. **b** shows the average fixation count for dentists viewing with (light blue) and without AI (dark blue). The black error bars represent a confidence interval at 95%.

Fixation frequency remained unaffected by AI support, fixations per second with AI support (2.21 [2.03, 2.33]) and without AI support (2.16 [1.98, 2.43]: *p* = 0.8287). Average fixation duration was also not significantly different between conditions (*p* = 0.7660), with median fixation durations at 313.27 ms [243.597, 367.13] (AI) and 317.29 ms [239.797, 349.86] (without AI). Figure 3 shows visual attention to relevant regions using fixation count to AOIs defined based on the user interface (AOIs illustrated in Fig. 3a). There were more fixations in the map and AI list of findings area when experts had the option for AI than without AI. Moreover, slight increases in the fixation counts on other AOIs related to the interface were apparent when AI was available (see Fig. 3b).

### Scanpath transitional behavior

**Fig. 4:**
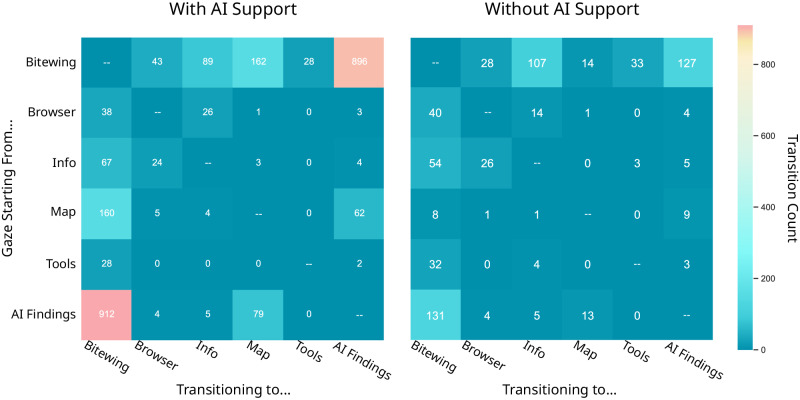
Gaze transitional behavior between AOIs with and without AI support. As we are interested in gaze transitions from one AOI to another, we exclude gaze transitions within an AOI as visualized by missing values along the diagonal. The warmer color tiles represent a higher number of gaze transition from one AOI to another AOI, read in the manner of from left to bottom.

We also looked at experts’ gaze transitions between AOIs when they have the option for AI support. Figure 4 reports the total number of transitions from each AOI to another AOI shown in Fig. 3a. Higher transitions are represented by warmer colors from one AOI to another AOI read in the manner of from left to bottom. With the AI support option, dentists had the highest gaze transitions from the AI list of findings to the bitewing (912 transitions) and the second-highest transitions being from bitewing back to the AI list of findings (896 transitions). The map of the teeth and bitewing also had a high number of transitions to and from. In general, fewer transitions were apparent when experts had no option for AI support, though interesting enough, the highest transitions were similar to the AI support condition, but at a much lower magnitude: 131 transitions from AI findings to bitewing and 127 transitions from bitewing to the AI findings. Without the AI support option, transitions to and from the tooth map were much less frequent, as the map was empty.

### User interface interactions

Even though AI support was optional, all dentists made use of the AI on the images where they were allowed to according to the randomization schedule. AI was turned on an average of 8.4 times (*range*: 1.1–19.5) and AI stayed on an average of 21012.05 ms (*range*: 9103.11–48506.51 ms).

**Fig. 5:**
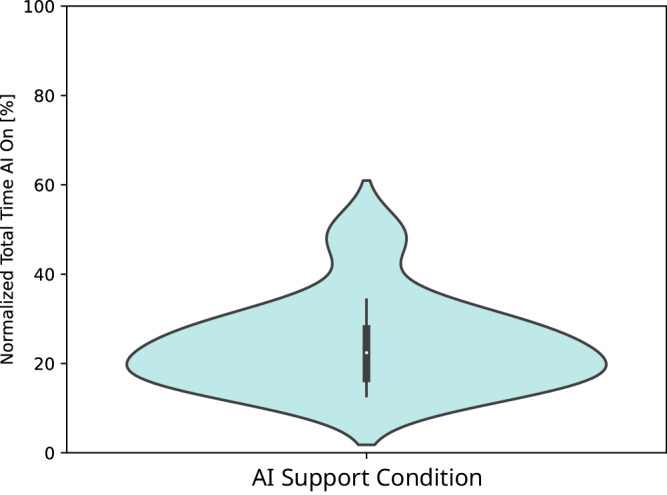
Total time participants had the AI support turned on, normalized to percent of their task time. In general, dentists turned the AI support on for less than half of the inspection time (around 25%, ranging from 12 to 50%).

Dentist on average turned the AI on after an average of 46793.94 ms (*sd* = 42731.65 ms), i.e. after the first 43.57 %(*sd* = 32.35 %) of task time. There was a higher number of fixations when they had the AI turned off (23.41 [15.98, 74.43]) compared to when they had the AI turned on (11.56 [4.94, 20.45]: *p* = 0.001), but this behavior can be attributed to them having the AI turned on for an average of 24.31 % of their total viewing time (Fig. 5). Average fixation duration slightly decreases when the user had AI support on (346.80 ms [264.57, 530.38]) compared to when they had it off (379.96 ms [282.37, 475.28]), though not significantly (*p* = 0.6781). Nor was fixation frequency significantly different when the user had AI support on (2.30 [1.92, 2.43]) compared to when they had it off (2.02 [1.76, 2.28]: *p* = 0.1784).

## Discussion

The aim of the present study was to use gaze analysis to observe how experts interact with an AI-based decision support tool to investigate dental bitewings. Although dentists’ time to inspect bitewings increased when they have the option to use AI support compared to normal visual inspection, the difference was not significant. However, their gaze behavior suggests they can accommodate to the increase in content from AI. These findings are decisive when considering that AI should set out to improve workflows^95^. Even the slightest increase in inspection time can evolve to large delays and fatigue in clinical environments.

Fixation metrics such as the fixation duration and frequency suggested that expert gaze behavior does not change when they have the option for AI support. Even in the context of when the AI was used, these metrics show no significant changes between when the AI is toggled on and off. From previous research, it is known that both these fixation metrics are affected by information content and extraction^96–100^. It would be expected that average fixation durations would increase to incorporate the additional visual overlays and interface information (tooth map and anomaly labels) the AI support offers. However, this was not the case for the current research. It has been found that fixation frequencies decreased to increasing uncertainty^101^ and increasing content density^100^. Thus, we expected lower fixation frequencies with AI support if the information the system provided would highlight areas dentists were unsure of. It seems that dental experts incorporated AI decision support information into their visual search strategies with little to no change from their usual (i.e, no AI support) visual inspection strategies.

However, there were significantly higher fixation counts when AI was available, especially attending to user interface elements containing AI support information. This finding aligns with usability research, where a higher number of fixations generally indicate more effort during the task^78,102^ or more components to investigate^103^. Dentists had nearly doubled their time spent attending to UI elements, which also is reflected in the higher fixation count on UI elements related to the AI support. Yet, fixation counts viewing the bitewing with AI support were only slightly higher than when there was no option for AI support. Similar behavior was found when using interactive AI systems for fact-checking^104^. Overall, dentists spent more time on task when AI support was available, which contributes to more fixations, but it does not affect the rate at which they visually processed the information, evident from the fixation duration and frequency metrics.

Regarding visual attention to specific elements of the system, aspects of the UI that were available for both conditions show almost equal attention (e.g., tools, info, and browser). However, there is a large increase in attention to the right side of the UI – where elements related to AI were – when there was the option for AI support. Regarding attentional transitions between the AOIs, there was an overall a higher number of transitions with AI support, mainly between the AI list of findings and the bitewing and between the tooth map and the bitewing. This behavior has implications that can be important for future designs of medical AI support systems, as these systems should not draw too much attention away from the medical image, which can increase inspection time and interrupt the already rapid and effective viewing patterns that experts have developed over their years of experience. Such long interruptions during expert visual inspection of CT scans have been found to increase inspection time, but not necessarily affect the diagnostic accuracy^105^. We can anticipate such behaviors, even if they are only slight disruptions, can build up over time and can contribute to fatigue.

**Fig. 6:**
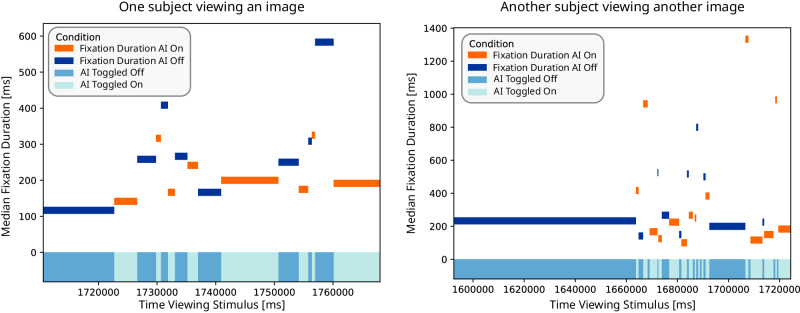
How experts interact with the AI support interface. When they have the AI turned off/ turned on is indicated in by the dark/light blue bars, respectively, at the bottom of the graph. The orange/navy blue bars are the median fixation durations during the respective on/off interval.

From our analysis, it seems that dentists employed the AI support as a *second reviewer*. Experts first investigated the image independently half of the task time, then used the AI, likely confirm their already made findings. Generally, they had the AI support turned on for almost 25% of the task time, but this behavior was varying, ranging from 12.72% to 50.04% of the time. Toggling the AI overlay on and off also varied between being turned on only once in one dataset up to 19 times in another dataset. Figure 6 shows two example interaction behaviors from two subjects for two different bitewings: One expert turned the AI on early and had it on longer than the average behavior (left subfigure), the other turned it on late and more frequently toggled it on and off (right subfigure). Fixation durations during these intervals also show quite the range, where overall durations were shorter when AI support is turned on (apparent in the right subfigure). It could be that experts adopt their own interaction styles, but further research is needed to confirm this behavior. Research that depicts AI as a second-reviewer system has promoted performance and human talents (e.g., creativity and heuristics) and has brought focus to system explainability^106–109^. Thus, finding the ideal harmony between user individuality and system information presentation – possibly when to offer suggestions and the reasoning behind it – can enhance interaction and even trust.

A limitation of the current work is that it does not address one of the most popular eye metrics for usability, the pupillary response as an indicator of task workload. This metric would have provided another level of understanding of mental effort with respect to AI support. The omission of pupillary response was needed, as we did not control for additional factors that can naturally reflect the pupillary reflex^110^. Pupillary response in experts has shown how experts accommodate increasingly difficult image information in medical image inspection^70^. Whether this behavior can also accommodate information presented by a decision support system has yet to be explored. Also, corroborating the visual attention with self reports towards the usability of the system would also provide a more direct link to professional opinion and potential improvements of the system. Since previous research has already established this link between gaze and usability^75–78^, we chose to focus on how gaze can allude to better interaction suggestions for future systems.Follow-up studies should consider standard usability questionnaires such as the System Usability Scale and User Experience Questionnaire after the experiment^111,112^. Performance was also not discussed in the current analysis, but in a detailed previous report^12^; dentists with AI support increased their sensitivity (without a decrease in specificity) for detecting caries compared to those without AI support. Lastly, acquiring data from more experts on more images in different environments would help promote the generalizability of our results.

Preliminary design suggestions from these findings could be offering the toggle option closer to the bitewing image, if not slightly overlaying it so as not to occlude relevant features. As experts employed the toggle option often, large transitions away from the main content to a small button on the side may not be favorable. Also, informational content, such as maps or reports, could be positioned closer to the relevant content (the bitewing), favoring shorter saccade lengths. Both suggestions need more research, though operate on a known design principle^113^. Finally, the ideal next steps of this research should include the factor of system explainability on how professionals visually interact with AI support systems. How error is communicated to users has shown varying effects on the interaction as well as trust in the system^114–116^. Eye movements can offer indicators for better ways to promote explainability without overloading the professional. We have shown that expert gaze behavior can help create better, more usable systems that are designed to promote the best abilities of both expert and AI, which ultimately benefits patient care.

## Methods

### Participants

Twenty-two dental experts (6 women, 16 men) volunteered to participate in the study. They were either employees at the dental hospital of Charité - Universitätsmedizin Berlin or worked in private practices in Berlin, Germany. Criteria for participation was having more than two years of clinical experience (i.e., had finished postgraduate education according to German insurance law), clinically active, and regularly detecting caries in their workflows (orthodontists and oral surgeons were excluded). All participants had normal to corrected vision.

### Materials

This research was part of a larger study encompassing dental professional performance with an AI decision-support system^12^ and their attention to specific lesions through the support of AI^117^. More specific details related to the task, the system and specific attention to dental features can be found in the previous studies^12,69^. There were two conditions that each participant experienced, bitewing inspection with AI support and without AI support, with all bitewings presented in a web browser that runs the AI software. As interacting with the software is the current research focus, we only briefly detail the bitewings, but^12^ provides further details on the bitewing content. From a database of 140 bitewings, 20 were randomly selected and presented in random order to each participant. Of these 20, ten bitewings were randomly selected to have the AI support available, which meant AI could be toggled on/off in the software. Due to the randomization process, bitewings were seen by multiple participants or in different conditions. All bitewings were of the permanent dentition, with at least the crowns of one jaw being visible. Each bitewing was checked and annotated for caries and restorations by four experts, with a fifth expert for crosschecking. A more detailed explanation of this labeling process can be found in ref. ^69^.

**Fig. 7:**
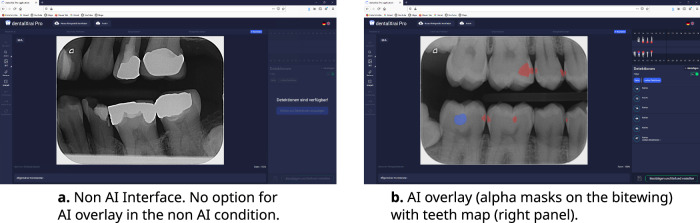
Example of web browser interface with and without AI overlays. Example of the web browser interface with presentation of the bitewing and left panel for image manipulations and right panel presented the AI information for the AI condition (**b**) or was blank for the non-AI condition (**a**).

The AI decision support system used was dentalXrai Pro 1.0.4, (dentalXrai Ltd, Berlin, Germany). This system is capable of fully automated AI X-ray reporting in everyday dental clinical practice. It detects pathologies and restorations, highlighting the findings in color, and automatically generates the written documentation. The software allowed the participant to view the native radiograph and its augmented version, where AI software detections are shown as pixel overlays on the bitewing. The participant could also add, remove, or change findings and generate an automated report. Participants in the AI condition can toggle the AI overlay on/off. Figure 7b shows an example of the software interface with the AI overlayed on the bitewing and the respective tooth mappings on the right panel. This example is what participants see when the AI is toggled on. The backbone of the software uses cloud-based machine learning to detect teeth, proximal carious lesions, and restorations visible on the bitewing images. For marking the teeth, a detection model based on U-Net^118^, whose findings had been validated by an experienced dentist for each bitewing image, was employed. The software version used for this study had a reported accuracy of 0.80, specificity of 0.75, and sensitivity of 0.83^119^. Further details of the algorithm and its performance can be found in ref. ^119^.

For consistency in presentation, the non-AI condition viewed images within the same web browser platform, but were not given the option to toggle the AI on and off. Figure 7a shows an example of the software for the non-AI condition. The stimulus remains almost the same for both conditions, but there is less information presented on the right side of the browser for the non-AI condition, as this region provided the AI content. Additionally, there are no pixel overlays on the bitewing images in this condition.

### Procedure

At least one week before the study, participants received a handbook about the AI software and were advised to try it out on a minimum of four independent bitewing radiographs. This was encouraged so that they had better understanding of the system and its capabilities. The study was run at either the dental hospital of Charité - Universitätsmedizin Berlin or the participant’s private practice.

The task evaluated for this current research was interacting with the software with AI support and without AI support, and how experts use the software to visually inspect images. 20 bitewing images were randomly chosen from the bitewing pool and presented in random order in the web browser that runs the dentalXrai software. This generation of images was unique to each participant. Prior to uploading an image into the software, each participant drew a slip of paper from a pool of 20 slips contained in a sealed opaque envelope (ten indicating to use the AI software and ten not) to determine which image would have the AI software (intervention) or not (control).

Participants performed the task in one session, operating at their own pace. They viewed the images in the web browser and depending on which condition the image was, they could use the AI software or could not. In the AI condition, dentists could then enable or disable the AI augmentations as needed. For both conditions, they then verbally reported any proximal caries detections and their corresponding treatment decisions to the study assistant, though this was not evaluated in the current analysis. The participants concluded the examination of the image, and the next one could be uploaded, following the protocol for drawing a slip of paper.

We chose this design method to be convenient for our participants. Asking medical professionals to set aside long or multiple windows of time can become harder for them to fit into their busy schedules. To avoid dropout rate or inconsistent lengths between two sessions, we chose one session. This choice can also control for errors in replicability of the setup, as we traveled to them. Additionally, one session, with highly randomized stimuli, better controls for any fatigue or learning effects participants may exhibit.

This evaluation is nested within a randomized, controlled, non-blinded, clustered cross-over, superiority trial with an allocation ratio of 1:1^12^, assessing the impact of an artificial intelligence (AI) software for detection of carious lesions. The trial was not conducted during clinical care and on actual patients, but on retrospectively sampled imagery material, which was randomly assessed with and without assistance from the AI software. The trial was registered at Deutsches Register Klinischer Studien (DRKS00022357). Ethical approval was provided by the Charité - Universitätsmedizin Berlin (EA/144/20). During the study, we had recorded dentists’ gaze patterns, and here we present the gaze pattern behavior of the control group (i.e., dentists not using AI). Written informed consent was obtained from all participating dentists.

### Eye tracking

To record gaze data, we used the SmartEye Aurora remote eye tracker running at 60 Hz, positioned under a monitor with a resolution of 1920 × 1080 pixels. Data collection took place in dimly lit rooms at either Charité or in private clinics for participant convenience; the study investigator brought the monitor to their clinic. Participants were unconstrained and sat approximately 70 cm from the tracker. An initial 9-point calibration and validation were performed. Recalibration was done if the software indicated that the calibration quality was poor. Gaze data was collected for the whole duration of the study using the *iMotions* software (version 8.2.22899.4). Event detection was performed using the *iMotions* implementation of the I-VT algorithm, with a minimum fixation duration of 60 milliseconds (ms) and a velocity threshold of 30deg/*s*. The current analysis used the fixations reported from the software, which are interpolated between the left and the right eye. We interpret fixations as the areas of attentional focus related to the stimuli presented on the screen.

### Data preparation

Data collection resulted in 445 datasets from the participants viewing bitewing radiographs. As five participants unintentionally examined one image twice, we excluded the first time they viewed the image, as it was too short for proper investigation (440 Datasets). To ensure gaze pattern data quality, we removed datasets with an average reported gaze signal quality lower than 0.60 (valid signal over total signal, using a scale of 0.0 being the lowest and 1.0 being the highest quality). 80 datasets were excluded from this criterion. A stimulus presentation error resulted in the removal of 11 further datasets. These exclusion criteria adhere to standard guidelines used in eye tracking research on data quality control^120,121^ Overall, 349 datasets (170 without AI and 179 with AI) were included in the current analysis.

### Reporting summary

Further information on research design is available in the Nature Research Reporting Summary linked to this article.

### Supplementary information

Reporting Summary

## Data Availability

The datasets used and/or analyzed during the current study are available from the corresponding author on reasonable request.

## Code availability

The code created and used for analyzing the preprocessed data for the current study is available from the corresponding author on reasonable request.
