# Supplementary material for: Expert gaze as a usability indicator of medical AI decision support systems: a preliminary study
Source: NPJ Digit Med. 2024 Jul 27;7:199. doi: 10.1038/s41746-024-01192-8 (PMC11283514; doi:10.1038/s41746-024-01192-8)
Supplement: Supplementary file 1 — Reporting Summary [file 41746_2024_1192_MOESM1_ESM.pdf]

nature portfolio

Corresponding author(s): Nora Castner

Last updated by author(s): Jun 25, 2024

Reporting Summary

Nature Portfolio wishes to improve the reproducibility of the work that we publish. This form provides structure for consistency and transparency in reporting. For further information on Nature Portfolio policies, see our Editorial Policies and the Editorial Policy Checklist.

Statistics

For all statistical analyses, confirm that the following items are present in the figure legend, table legend, main text, or Methods section.

n/a	Confirmed	
☐	☒	The exact sample size (*n*) for each experimental group/condition, given as a discrete number and unit of measurement
☐	☒	A statement on whether measurements were taken from distinct samples or whether the same sample was measured repeatedly
☐	☒	The statistical test(s) used AND whether they are one- or two-sided<br>*Only common tests should be described solely by name; describe more complex techniques in the Methods section.*
☒	☐	A description of all covariates tested
☐	☒	A description of any assumptions or corrections, such as tests of normality and adjustment for multiple comparisons
☐	☒	A full description of the statistical parameters including central tendency (e.g. means) or other basic estimates (e.g. regression coefficient) AND variation (e.g. standard deviation) or associated estimates of uncertainty (e.g. confidence intervals)
☐	☒	For null hypothesis testing, the test statistic (e.g. *F*, *t*, *r*) with confidence intervals, effect sizes, degrees of freedom and *P* value noted<br>*Give P values as exact values whenever suitable.*
☒	☐	For Bayesian analysis, information on the choice of priors and Markov chain Monte Carlo settings
☒	☐	For hierarchical and complex designs, identification of the appropriate level for tests and full reporting of outcomes
☒	☐	Estimates of effect sizes (e.g. Cohen's *d*, Pearson's *r*), indicating how they were calculated

Our web collection on statistics for biologists contains articles on many of the points above.

Software and code

Policy information about availability of computer code

Data collection	The AI decision support system used was dentalXrai Pro 1.0.4, (dentalXrai Ltd, Berlin, Germany). To record gaze data, we used the SmartEye Aurora remote eye tracker running at 60 Hz, positioned under a monitor with a resolution of 1920 × 1080 pixels.
Data analysis	Gaze data was collected for the whole duration of the study using the iMotions software (version 8.2.22899.4). Event detection was performed using the iMotions implementation of the I-VT algorithm, with a minimum fixation duration of 60 milliseconds (ms) and a velocity threshold of 30deg/s. The current analysis used the fixations reported from the software, which are interpolated between the left and the right eye. All statistical analyses and data management were performed using Python (version 3.8 and above).

For manuscripts utilizing custom algorithms or software that are central to the research but not yet described in published literature, software must be made available to editors and reviewers. We strongly encourage code deposition in a community repository (e.g. GitHub). See the Nature Portfolio guidelines for submitting code & software for further information.

Data

Policy information about availability of data

All manuscripts must include a data availability statement. This statement should provide the following information, where applicable:

- Accession codes, unique identifiers, or web links for publicly available datasets
- A description of any restrictions on data availability
- For clinical datasets or third party data, please ensure that the statement adheres to our policy

The datasets used and/or analyzed during the current study are available from the corresponding author on reasonable request.

Research involving human participants, their data, or biological material

Policy information about studies with human participants or human data. See also policy information about sex, gender (identity/presentation), and sexual orientation and race, ethnicity and racism.

Reporting on sex and gender	Twenty-two dental experts (6 women, 16 men) volunteered to participate in the study. Sex and Gender and their effects on Interaction with the software were not investigated in this research
Reporting on race, ethnicity, or other socially relevant groupings	For privacy preserving reasons, we did not ask participants to provide personal information regarding race, ethnicity, or other socially relevant groupings.
Population characteristics	Participants were either employees at the dental hospital of Charité – Universitätsmedizin Berlin or worked in private practices in Berlin, Germany. Criteria for participation was having more than two years of clinical experience (i.e., had finished postgraduate education according to German insurance law), clinically active, and regularly detecting caries in their workflows (orthodontists and oral surgeons were excluded). All participants had normal to corrected vision.
Recruitment	Recruitment of participants took place between October 2020 and January 2021. The sample of dentists was limited and selected; the sample was younger than the average German dentist and mainly situated in an urban clinic or practice environment. As laid out in the paper, this potentially affected the results such that the participants were more accurate than what could be expected.
Ethics oversight	The trial was registered at Deutsches Register Klinischer Studien (DRKS00022357). Ethical approval was provided by the Charité – Universitätsmedizin Berlin (EA/144/20).

Note that full information on the approval of the study protocol must also be provided in the manuscript.

Field-specific reporting

Please select the one below that is the best fit for your research. If you are not sure, read the appropriate sections before making your selection.

☐ Life sciences ☒ Behavioural & social sciences ☐ Ecological, evolutionary & environmental sciences

For a reference copy of the document with all sections, see nature.com/documents/nr-reporting-summary-flat.pdf

Behavioural & social sciences study design

All studies must disclose on these points even when the disclosure is negative.

Study description	This evaluation is nested within a randomized, controlled, non-blinded, clustered cross-over, superiority trial with an allocation ratio of 1:112, assessing the impact of an artificial intelligence (AI) software for detection of carious lesions. The trial was not conducted during clinical care and on actual patients, but on retrospectively sampled imagery material, which was randomly assessed with and without assistance from the AI software
Research sample	Expert dentists that either employees at the dental hospital of Charité – Universitätsmedizin Berlin or worked in private practices in Berlin, Germany.
Sampling strategy	We chose this design method to be convenient for our participants. Asking medical professionals to set aside long or multiple windows of time can become harder for them to fit into their busy schedules. To avoid dropout rate or inconsistent lengths between two sessions, we chose one session. This choice can also control for errors in replicability of the setup, as we traveled to them. Additionally, one session, with highly randomized stimuli, better controls for any fatigue or learning effects participants may exhibit.
Data collection	Data collection employed a computer and monitor setup running the AI-software (dentalXrai Pro 1.0.4, (dentalXrai Ltd, Berlin, Germany) and an eye tracker (SmartEye Aurora remote eye tracker, 60 Hz, powered by the software iMotions, version 8.2.22899.4) Pen and paper were used for recording notes, though this was not evaluated in the study. There were two conditions that each participant

experienced, bitewing inspection with AI support and without AI support, with all bitewings presented in a web browser that runs the AI software. As interacting with the software is the current research focus, we only briefly detail the bitewings, but12 provides further details on the bitewing content. From a database of 140 bitewings, 20 were randomly selected and presented in random order to each participant. Of these 20, ten bitewings were randomly selected to have the AI support available, which meant AI could be toggled on/off in the software. Due to the randomization process, bitewings were seen by multiple participants or in different conditions. All bitewings were of the permanent dentition, with at least the crowns of one jaw being visible. Each bitewing was checked and annotated for caries and restorations by four experts, with a fifth expert for crosschecking.

Timing	Data collection took place between October 2020 and January 2021.
Data exclusions	Criteria for participation<br>was having more than two years of clinical experience (i.e., had finished postgraduate education according to German insurance law), clinically active, and regularly detecting caries in their workflows (orthodontists and oral surgeons were excluded). We further carefully detail our process for exclusion of certain corrupt gaze data. We follow common and standardized steps for controlling for signal errors which is common in eye tracking studies
Non-participation	No Participants dropped out during participation
Randomization	Order of AI/Non AI was randomized for each participant and which image present in either condition was randomized. As experts have very busy schedules, 20 bitewing images were randomly chosen from the bitewing pool (of 140 images)<br>and presented in random order in the web browser that runs the dentalXrai software. This generation of images was unique to each participant. Prior to uploading an image into the software, each participant drew a slip of paper from a pool of 20 slips contained in a sealed opaque envelope (ten indicating to use the AI software and ten not) to determine which image would have the AI software (intervention) or not (control). Asking medical professionals to set aside long or<br>multiple windows of time can become harder for them to fit into their busy schedules. To avoid dropout rate or inconsistent lengths between two sessions, we chose one session. This choice can also control for errors in replicability of the setup, as we traveled to them. Additionally, one session, with highly randomized stimuli, better controls for any fatigue or learning effects participants may exhibit.

Reporting for specific materials, systems and methods

We require information from authors about some types of materials, experimental systems and methods used in many studies. Here, indicate whether each material, system or method listed is relevant to your study. If you are not sure if a list item applies to your research, read the appropriate section before selecting a response.

Materials & experimental systems

n/a	Involved in the study	
☒	☐	Antibodies
☒	☐	Eukaryotic cell lines
☒	☐	Palaeontology and archaeology
☒	☐	Animals and other organisms
☒	☐	Clinical data
☒	☐	Dual use research of concern
☒	☐	Plants

Methods

n/a	Involved in the study	
☒	☐	ChIP-seq
☒	☐	Flow cytometry
☒	☐	MRI-based neuroimaging

Plants

Seed stocks	n/a
Novel plant genotypes	n/a
Authentication	n/a
